# Improving work outcomes in people with multiple sclerosis: A scoping review of non-pharmacological interventions

**DOI:** 10.1177/02692155261454943

**Published:** 2026-05-25

**Authors:** Shalina RD Saddal, Blanca De Dios Perez, Alejandra Machado, Emilie Friberg, Roshan das Nair, Kathryn Radford, Joost G Daams, Hanneke E Hulst, Vincent de Groot, Karin van der Hiele, Frederieke Schaafsma

**Affiliations:** 1 MS Center Amsterdam, Public and Occupational Health, Vrije Universiteit Amsterdam, Amsterdam Public Health Research Institute, 1209 Amsterdam UMC location VUmc, Amsterdam, The Netherlands; 2 Health, Medical and Neuropsychology Unit, Institute of Psychology, Faculty of Social Sciences, 4496 Leiden University, Leiden, The Netherlands; 3 MS Center Amsterdam, Rehabilitation Medicine, Vrije Universiteit Amsterdam, Amsterdam Neuroscience, Amsterdam UMC location VUmc, Amsterdam, The Netherlands; 4 Public and Occupational Health, Amsterdam Public Health Research Institute, Amsterdam UMC location VUmc, Amsterdam, The Netherlands; 5 Centre for Rehabilitation & Ageing Research, School of Medicine, 6123 University of Nottingham, Nottingham, UK; 6 574111 NIHR Nottingham Biomedical Research Centre, Nottingham, UK; 7 Division of Insurance Medicine, Department of Clinical Neuroscience, 27106 Karolinska Institutet, Stockholm, Sweden; 8 Mental Health & Clinical Neurosciences, 6298 University of Nottingham, Nottingham, UK; 9 Department of Health, SINTEF, Trondheim, Norway; 10 Central Unit for Research, Innovation, and Education in Mental Health, Clinic of Mental Healthcare, 60510 St Olavs Hospital, University Hospital Trondheim, Trondheim, Norway; 11 Medical Library, 26066 Amsterdam UMC location AMC, Amsterdam, The Netherlands; 12 School of Psychology, Faculty of Science, University of Auckland, Auckland, New Zealand; 13 Psychological Medicine, Faculty of Health and Medical Sciences, University of Auckland, Auckland, New Zealand; 14 Center or Brain Research, University of Auckland, Auckland, New Zealand

**Keywords:** Multiple sclerosis, employment, work outcomes, non-pharmacological intervention, review

## Abstract

**Objective:**

To evaluate the effectiveness of non-pharmacological intervention studies on work outcomes in people with multiple sclerosis (MS).

**Data sources:**

We searched three electronic databases (MEDLINE, PsycINFO, and Web of Science: Conference Proceeding Citation Index) and included studies reporting on having/returning to paid employment, work productivity, or factors related to work ability.

**Review methods:**

The Cochrane Handbook for Systematic Reviews of Interventions and the PRISMA extension for scoping reviews checklist were followed. All steps were conducted by two reviewers.

**Results:**

The search (inception-April 2026) yielded 10449 articles. Sixteen studies were included: four vocational, seven psychological, one exercise, and four combining these intervention types. Reported work outcomes included employment status, work difficulties, workplace accommodations, promotions, impact on work and education, work productivity and impairment, and work and social adjustment. Eight studies aimed to improve work outcomes. Five interventions demonstrated significant improvement: two targeting fatigue with cognitive behavioral therapy (β = −0.34, 95% CI: −0.66 to −0.03; β = −3.68, 95% CI: −5.82 to −1.537), one on conscientiousness (effect size not reported), one on information processing speed (*f*2 = 0.04, *p* = 0.044), and one on job seeking (career goals and appraisal of potential work difficulties (*t* = −2.68, *p* = 0.009), self-esteem (*t* = 2.47, *p* = 0.016), relations (*t* = 2.44, *p* = 0.017), finances (*t* = 2.14, *p *= 0.036)).

**Conclusion:**

Few non-pharmacological interventions evaluate work outcomes. Cognitive behavioral therapy targeting MS-related fatigue shows promise for improving work outcomes. The heterogeneity of MS and variability in work contexts add challenges, indicating that personalized interventions may ultimately prove more effective than generic approaches.

## Introduction

Multiple sclerosis (MS) is the most prevalent neurological disorder in adults of working age, with over 2.8 million cases globally.^1^ Unemployment in people with MS often occurs in the first three to ten years after diagnosis.^2^ People with MS experience work disability due to a combination of disease-related (e.g. fatigue, physical disability, and cognitive problems),^3–5^ work-related (e.g. workplace inflexibility), and personal (e.g. education level)^6,7^ factors. Unemployment negatively impacts the quality of life in people with MS.^8,9^ Moreover, employment is a human right and important from a: (i) personal perspective (financial, general well-being, life purpose, physical and mental health^10,11^), (ii) family perspective (life partners of unemployed people with MS experience a higher level of caregiver strain than those of people with MS with a paid job^12^), (iii) societal perspective (work participation is needed for the economic and social demands of an ageing society, and those who remain at work use fewer social benefits and contribute to taxes^13,14^). Employed people with MS report more negative work experiences, more work difficulties, decreased work functioning, and higher work absence and presenteeism than employees without MS.^15,16^ Thus, both unemployment and work-related problems have a large impact on people with MS, their working and private environment and society in general.

Because of the negative impact of unemployment and problems experienced by people with MS at work, interventions that support job retention and help manage work problems have been developed. These are commonly known as “vocational rehabilitation.” The last review evaluating the effectiveness of vocational rehabilitation interventions, published in 2009, concluded that there was inconclusive evidence to support these interventions for people with MS.^17^ Since then, developments in the employment field have impacted job opportunities for people with chronic conditions, such as MS. For instance, the rise of digital tools and applications,^18^ as well as changes in the structure of the workplace since the COVID-19 pandemic, have led to remote or hybrid working, regular breaks and flexible working hours.^19^ The increasing attention to social participation and employment in scientific research has also impacted MS research.^20,21^ Several recent studies examining the effectiveness of interventions aimed at managing MS symptoms, also included (secondary) work outcomes. This creates an opportunity to study the effectiveness of other interventions, besides vocational rehabilitation interventions, on work-related outcomes, which have not been reviewed before.

This scoping review aimed to identify non-pharmacological interventions for people with MS, specifically vocational-, psychological-, and exercise interventions, including a work-related outcome and synthesize the evidence of their effectiveness in supporting work outcomes.

## Methods

We conducted a scoping review to map the wider breadth of existing evidence on interventions supporting work outcomes, instead of focusing on the effectiveness of specific interventions (e.g. Vocational Rehabilitation). Additionally, we synthesized the evidence of their effectiveness in improving work outcomes. We used the PRISMA extension for scoping reviews checklist^22^ and the Cochrane Handbook For Systematic Reviews of Interventions^23^ to design and implement the study and present the findings according to our previously published protocol.^24^ Furthermore, we aimed to ensure a high-quality review by following extensive rigor during all stages of the review.

Electronic databases were searched from inception to August 2024 by an experienced librarian (JD) in Medline, PsycINFO, and Web of Science: Conference Proceedings Citation Index (Supplemental material 1). An updated search was performed in April 2026. We searched for published results of protocol papers and scanned the reference lists of relevant reviews and other relevant ineligible studies identified with the search. Authors of articles that included a subset of people with MS were contacted to check whether data were available for the MS participants.

We selected English language randomized controlled trials and non-randomized controlled trials with adults in paid employment or considering moving into paid employment, with a confirmed MS diagnosis. Studies in a sub-group of MS patients (e.g. fatigued people with MS or cognitively impaired people with MS) were not excluded.

Selected studies evaluated the effectiveness of non-pharmacological interventions, by comparing these to an alternative (control) intervention. Pharmacological interventions (which have developed considerably over the last 10 years^25^ and significantly affect employment in people with MS^26^) and other non-pharmacological intervention types (e.g. mindfulness or diet interventions) may also impact work outcomes. However, for feasibility and pragmatic reasons, we limited the scope of the current review to three specific non-pharmacological intervention types: vocational-, psychological-, and exercise interventions.

Outcomes of interest were work outcomes in the following stages of work participation: in paid employment, return to paid employment, increasing or maintain work productivity, and factors related to work ability. The predefined work outcomes were selected based on the core outcome set for work as defined by Ravinskaya and colleagues,^27,28^ which describes what work outcomes are relevant under these four overarching outcome categories.

Additional work outcomes that did not fit within this scope were included in an “other” category. Abstracts and titles were screened by five authors (SS, BDP, KvdH, AM, FS). The full text was screened by SS, KvdH, AM, and FS. Each action was conducted by two independent reviewers. Disagreements were solved by a third independent reviewer (VG). Reviews, qualitative studies, and protocol papers were checked for relevant references by one author (SS). The full methods are described in the protocol,^24^ published on dataverseNL on 16 July 2024.

Two authors (SS, BDP) independently assessed the risk of bias for each study. The RoB2 from the Cochrane Handbook for Systematic Reviews of Interventions^29^ was used to assess the risk of bias of randomized controlled trials, and the ROBINS-I-V2 tool^30^ was used for non-randomized studies. Disagreements were resolved by these authors through a consensus meeting.

A data extraction form was developed, and five reviewers (SS, BDP, KR, FS, KvdH) independently extracted the data for each study. A third reviewer (RdN) randomly selected two papers (representing approximately 20% of the total included), read them first, and checked the data entry against the information in the papers. Where any discrepancies arose, queries were raised with the data extractors.

The effectiveness of the included studies was discussed per intervention type. Quantitative data were analyzed using descriptive methods and narratively synthesized.

## Results

The PRISMA flow diagram (Figure 1) shows identified, screened, and included articles for both the initial and updated searches. The searches resulted in a total of 10449 records, of which 1134 duplicates were removed before screening. The titles and abstracts of 9315 records were screened against the inclusion criteria, of which the full text was retrieved for 466 articles. Of these, 13 articles were included in the review. Thirty seven articles were considered for eligibility based on full text screening of relevant reviews, qualitative studies and protocols. Three additional studies were included in the review through other methods. A total of 16 studies met the inclusion criteria and were included in this scoping review.

**Figure 1.**
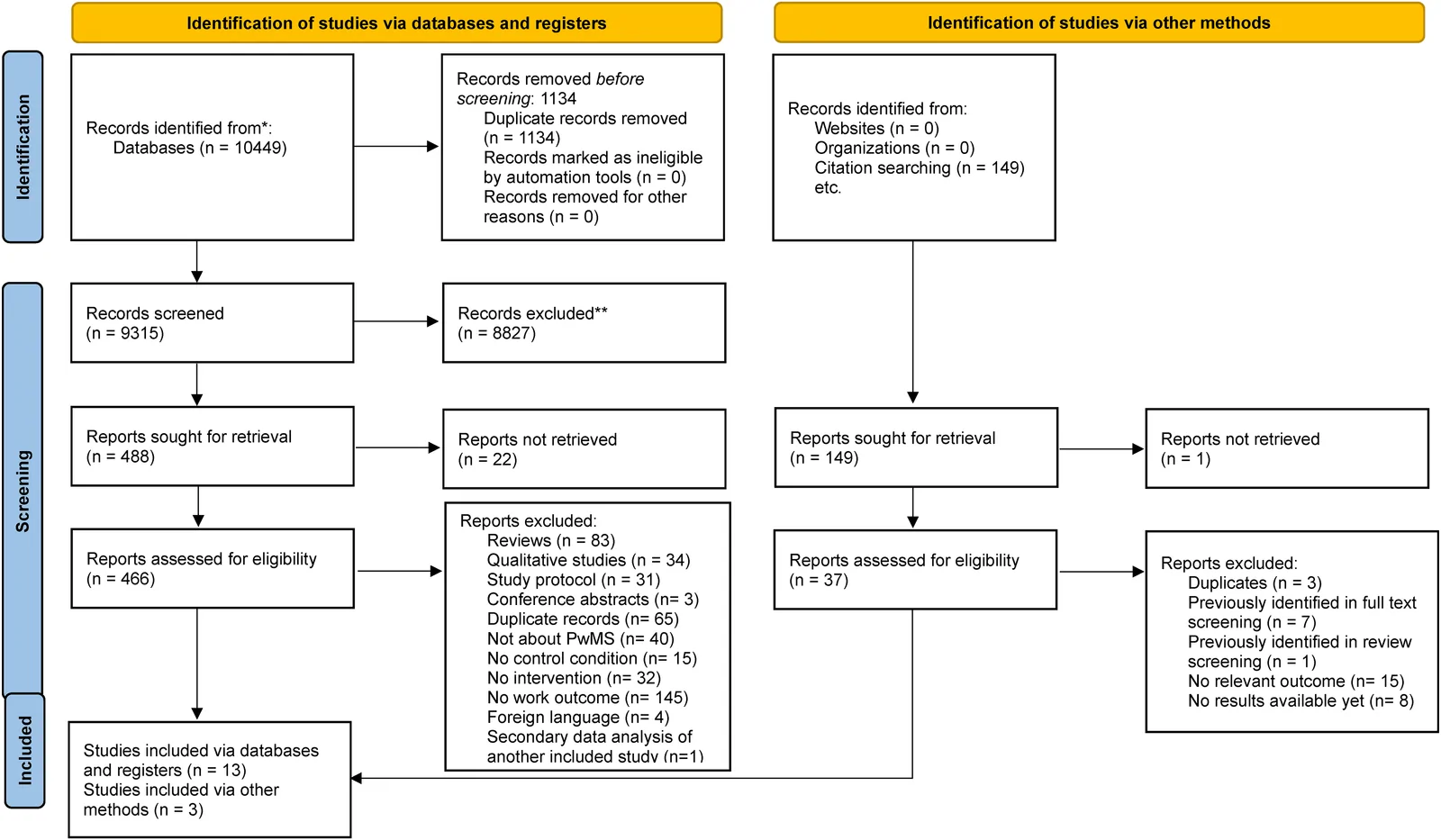
Flow diagram (PRISMA 2020).

Overall, only minor queries arose from the initial spot check on the data extraction forms, so no further spot checks were necessary. We decided against quantifying these because the number of data entry points varied between papers (based on what was extractable) and the “weight” of each datapoint extracted was not the same (for instance, a typo related to the date of publication is not the same as one related to an effect size).

The initial agreement between the two assessors in risk of bias assessments was 81%. No disagreements remained after a consensus meeting. Figure 2 depicts an overview of the risk of bias assessments of the randomized controlled trial studies. For the non-randomized controlled trial study^31^ risk of bias was assessed with the ROBINS-I-V2 tool, therefore the risk of bias assessment of this study is not included in the figure. This study was classified as having some concern. Overall, seven studies were classified as having a high risk of bias,^20,32–37^ eight as having some concern,^31,38–44^ and one as having a low risk of bias.^45^

**Figure 2.**
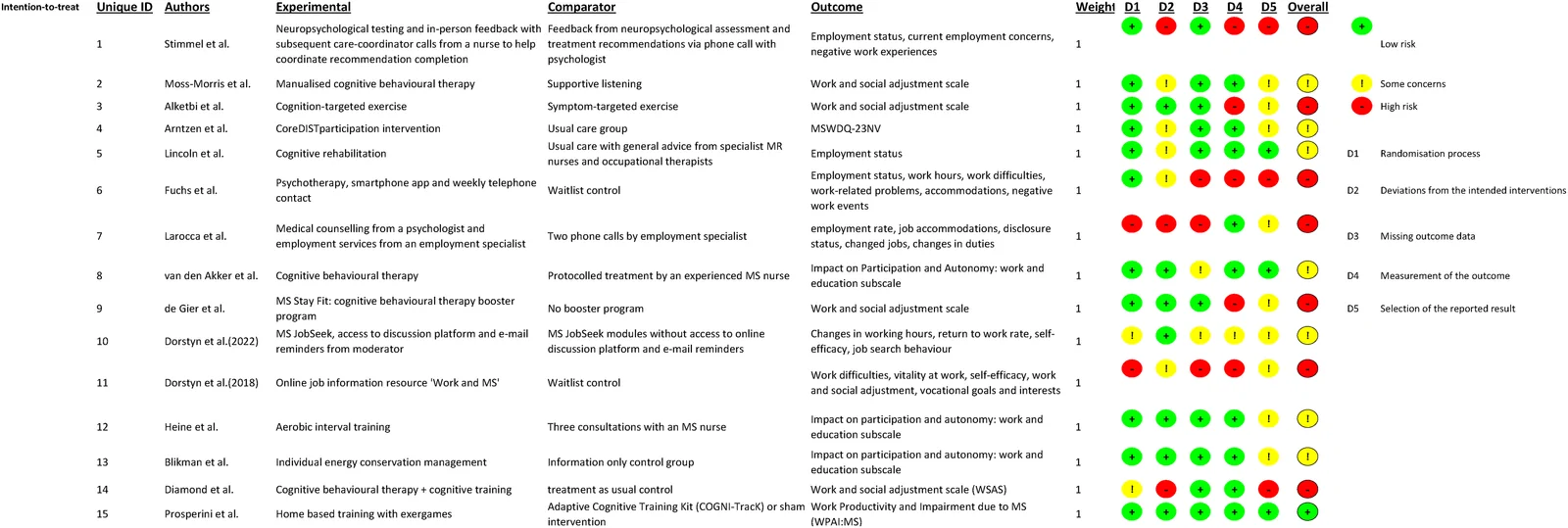
Risk of bias RoB2.

Included studies were published between 1996 and 2026 and used the following designs: randomized controlled trial (n = 12^20,34–38^^,40–45^), pilot randomized controlled trial (n = 2^33,39^), quasi-RCT (n = 1^32^), and a non-randomized controlled trial (n = 1^31^). Most studies were conducted in the United States (n = 4; 29%^31,32,34,35^) and the Netherlands (n = 4; 29%^36,41,43,44^). Sample sizes ranged between 11^34^ and 449^40^ participants. Across studies, participants were, on average, 45.3 years old, and 77% were women. Studies reported, on average, 14.7 years of education, with most participants having a degree or diploma of medium to high education. Four studies did not report educational level.^39,41,43,44^ Ethnicity was reported in only three studies,^32,38,40^ all of which had predominantly white participant samples.

In all studies that reported MS type (n = 11^20,33,34,36–45^), at least 65% of participants had relapsing remitting MS. Thirteen studies reported disease duration, with an average of 12.7 years across studies that reported means (n = 7^31,33–35^^,37,39,40,45^), and an average of 6.9 years across studies that reported median duration (n = 6^36,38,41–44^). Four studies did not report any (neuro)psychological problems.^20,34,39,42^ Eight studies reported presence of fatigue,^31–33^^,36,40,41,43,44^ five reported subjective cognitive problems,^31,35,40,43,44^ three reported cognitive disorders,^32,38,40^ and two studies reported anxiety or depression.^37,38^ Three studies did not report on the employment situation at baseline.^37,38,44,45^ In studies that reported on the employment situation (n = 10), employment rates ranged between 0%^31^ and 100%.^34^

The interventions were classified as vocational interventions^20,31,35,42^ (n = 4), psychological interventions^34,36–38^^,40,41,44^ (n = 7), exercise interventions^43^ (n = 1), and combined interventions (n = 4) (vocational and psychological,^32^ vocational and exercise,^39^ psychological and exercise^33,45^). There was considerable heterogeneity among intervention types and outcomes measured. In addition, the target group differed depending on the focus of the intervention (e.g. severely fatigued people with MS, women with MS). Only eight of these studies primarily aimed to improve work outcomes.

An overview of studies evaluating the effectiveness of vocational interventions^20,31,35,42^ on work outcomes for people with MS is depicted in Table 1. In these four studies, the work-related outcome was also the primary outcome.

**Table 1.** Summary of vocational interventions.

First author, year, country	Study design Length of follow-up	Work-related outcome measure	Target group (T) Intervention (I) control (C)/ Alternative intervention (A)	Sample characteristics: demographic factors, MS features, (neuro)psychological problems, employment situation	Effectiveness of the intervention on employment outcome(s)
*Vocational interventions*					
Larocca et al., 1996^35^, USA*	RCT 1 year	Employment rate (part/fulltime) Job accommodations Disclosure status Changed jobs Changes in duties	**T:** Employed PwMS at risk of losing their jobs (self-report). **I:** Medical counselling from a psychologist and employment services from an employment specialist (two 1-h visits at the MS-center). **A:** Two phone calls by employment specialist at approximately 12-month interval.	**N** = 41 **Age M(SD):** 41.6(9.6) **Women:** 75.6%, **Men:** 24.4% **Years of education M(SD):** 14.9(2.4) **Ethnicity:** NR **MS type:** NR **Disease duration M(SD):** duration of symptoms 10.7(8.3) years **(Neuro)psychological problems:** 6 reported cognitive problems **Employment situation:** 41 (all) participants were employed, at risk for job loss. 83% full-time, 9.7% parttime, 7.3% on leave (not reported whether this was sick-leave or holidays) Disclosure to employer: 9.8% no, 90.2% yes	No significant differences in employment rate between I and A groups. Four persons in I and three in A had obtained new accommodations during the project. Two persons in I had not disclosed their MS at entry but had done so by the end of the 12-month follow-up. Of the two persons in A that had not disclosed, one had done so by 12 months. In both I and A there were few changes in employment during the 12 months of the project. One person in I changed jobs, none in A did so. Three persons in I, and two in A had some significant change in their duties.
Rumrill et al., 1998^31^, USA*	NRCT 16 weeks	Employment status (RTW) Accommodation self-efficacy Employability Maturity interview Job seeking activities	**T:** Unemployed PwMS. **I:** Job placement program: Matching a local employer and vocational rehabilitation counsellor, accommodations planning team (APT) seminar and telephone and in-person follow-along support. **A:** More traditional job seeking skills intervention.	**N** **=** 37 **Age M(SD):** 43(-) **Women:** 78%, **Men:** 22% **Years of education**: +- 14 on average **Ethnicity:** NR **MS type:** NR **Disease duration M(SD):** 12(-) years **(Neuro)psychological problems:** fatigue and cognitive problems (no numbers stated) **Employment situation:** 37 (all) unemployed	No significant differences in work-related outcomes between I and A groups.
Dorstyn et al., 2018^20^, Australia*	RCT 4 weeks	Work difficulties (MSWDQ) Vitality at work (optimism) Self-efficacy Work and social adjustment Vocational goals and interests	**T:** Community-dwelling adults (18 years or older) with MS actively seeking employment. **I:** Online Job information resource ‘Work and MS’. Intervention participants were e-mailed the first 3 Work and MS modules (focus on the job-seeking process) to review at their own pace. A follow-up email with the remaining 4 modules (focus on job-interviewing skills and career development) was sent 1 week later. **C:** After completion of the questionnaires at 4-weeks follow-up, control participants were e-mailed the Work and MS resource.	**N** **=** 95 **Age M(SD):** 41.3(9.8) **Women:** 85%, **Men:** 15% **Education level:** 4% some high school, 18% high school completion, 63% degree or diploma, 15% other. **Ethnicity:** NR **MS type:** 88% RRMS, 1% PRMS, 5% SPMS, 1% PPMS **Disease duration:** NR **(Neuro)psychological problems:** - **Employment situation:** 77% employed yet seeking alternative work. Work classification = working or not working, yet want to.	Significant improvement in I group on ‘my vocational situation scale’(*t* = −2.68, *p* = 0.009), “MSWDQ self-esteem”(*t* = 2.47, *p* = 0.016), “MSWDQ relations” (*t* = 2.44, *p* = 0.017), “MSWDQ finances” (*t* = 2.14, *p* = 0.036) No significant differences in “job procurement self-efficacy scale,” “MSWDQ work-home balance,” “MSWDQ accessibility,” “MSWDW supports” between I and C groups.
Dorstyn et al., 2022^42^, Australia & New Zealand*	RCT 4 months	Changes in working hours Return to work rate (part/full time) Self-efficacy Job search behavior	**T:** Adults of working age with relapsing-remitting or progressive forms of MS. **I:** MSJobSeek included modules covering job-searching and planning skills including several built-in features to promote engagement. In addition, intervention participants got access to a discussion platform with activity feed notifications and the possibility to interact. E-mail from moderator if they did not log in for more than one week. **A:** The same modules that were available on the MS JobSeek forum for intervention participants to view and download were available for the control group, without access to the online discussion platform and e-mail reminders from moderator.	**N** **=** 29 **Age M(SD):** 45.4(9.5) **Women:** 75.9%, **Men:** 24.1% **Education level:** 3.5% some high school, 10.3% completed high school, 27.6% post-secondary diploma or certificate, 34.5% undergraduate degree, 24.1% postgraduate degree **Ethnicity:** NR **MS type:** 79.3% RRMS, 17.2% SPMS, 3.5% PPMS **Disease duration median months(IQR):** 115(49–180) **(Neuro)psychological problems**: - **Employment situation:** 86.2% unemployed, 13.8% employed	No significant differences in work-related outcomes between I and A groups.

MS: multiple sclerosis; PwMS: people with MS; RCT: randomized controlled trial; NRCT: non-randomized controlled trial; RTW: return to work; RRMS: relapsing-remitting MS; T: target group; I: intervention group; C: control group; A: alternative intervention group; M: mean; SD: standard deviation; NR: not reported; *: primarily aimed to improve work outcomes.

Larocca et al.^35^ studied the feasibility of a job-retention intervention for improving employment rate and did not find significant differences between the intervention and control groups. Rumrill et al.^31^ compared the effectiveness of a job placement program to a more traditional job seeking skills intervention for improving employment status, accommodation self-efficacy, employability, maturity interview, and job seeking activities in people with MS. No significant differences were found in work-related outcomes between intervention and alternative intervention groups. Dorstyn et al.^20^ evaluated the effectiveness of an online resource for job seekers with MS for improving work difficulties, vitality at work, self-efficacy, work and social adjustment, and vocational goals and interest. The intervention led to improved confidence in career goals (*t* = −2.68, *p* = 0.009) and more positive appraisal of potential work difficulties (self-esteem (*t* = 2.47, *p* = 0.016), relations (*t* = 2.44, *p* = 0.017), finances (*t* = 2.14, *p* = 0.036)) in the intervention group (online job seeking modules) compared to the waitlist control group. Dorstyn et al.^42^ studied the effectiveness of an online peer discussion forum to improve exploration of employment seekers with MS. The intervention and control groups did not significantly differ on changes in working hours, return to work rate, self-efficacy, and job search behavior.

The studies evaluating the effectiveness of psychological interventions^34,36,38,40,41,44^ on work outcomes are depicted in Table 2*.* In two of these seven studies,^34,44^ the primary outcome was work-related, whereas five studies^36–38^^,40,41^ included a work outcome as a secondary outcome.

**Table 2.** Summary of psychological interventions.

First author, year, country	Study design Length of follow-up	Work-related outcome measure	Target group (T) Intervention (I) control (C)/ Alternative intervention (A)	Sample characteristics: demographic factors, MS features, neuropsychological/psychological problems, employment situation	Effectiveness of the intervention on employment outcome(s)
*Psychological interventions*					
Moss-Morris et al., 2013^38^, UK	RCT 12 months	Work and social adjustment (WSAS)	**T:** PwMS within 10 years of diagnosis. **I:** manualized CBT consisting of nine chapters, with activities and homework sheets, which could be individualized to the participant needs. Participants worked with a nurse therapist to develop a formulation of their particular areas of strengths and difficulties, decided on areas to focus on, and set tasks or homework to do between sessions. Eight individual sessions over a ten-week period. The first six sessions were scheduled weekly and face-to-face (80–90 min long) and the last two fortnightly (by telephone, up to 1 h long). **A:** Supportive listening (SL): participants were given the opportunity to talk freely and confidentially about their experiences, thoughts, and feelings about MS and its effect on their lives.	**N** **=** 94 **Age M(SD):** 41.8(9.54) **Women:** 69%, **Men:** 31% **Education level:** 73% A levels or higher **Ethnicity:** I: 79.2% white British, C: 71.7% white British. **MS type:** 78% RRMS, 10% SPMS, 12% PPMS **Disease duration median:** 3 years (range 0.8–10 years) **(Neuro)psychological problems:** 47.1% anxiety or depression. **Employment situation:** NR.	No significant differences in WSAS between I and A groups. WSAS scores tended to be lower in I than A at the end of therapy (β = −0.22, 95% CI: −4.37 to 0.84).
Lincoln et al., 2020^40^, UK	RCT 1 year	Employment status	**T:** PwMS aged 18–69 years, who reported cognitive problems in daily life and had cognitive problems on standardized assessment. **I:** cognitive rehabilitation provided by an assistant psychologist to groups of four to six participants, who met approximately weekly for ten sessions. The intervention included restitution strategies to retrain attention and memory functions and strategies to improve coding and retrieval. **C:** usual care with general advice from specialist MS nurses and occupational therapists on how to manage any cognitive difficulties.	**N** **=** 449 **Age M(SD):** 49.4 (9.9) **Women:** 73%, **Men:** 27% **Years of education M(SD):** 14.1(3.15) **Ethnicity:** I: 97% white, C: 96% white. **MS type:** 65% RRMS, 25% SPMS, 10% PPMS **Disease duration M(SD) years:** 11.6(8.35) **(Neuro)psychological problems:** Fatigue Severity Scale M(SD): 1.4(1.4), 100% subjective cognitive problems, 100% cognitive disorders. **Employment situation:** 151 employed or in education, 297 unemployed.	No significant differences in employment status between I and C groups.
Fuchs et al., 2022^34^, USA*	RCT 12 weeks	Employment status Work hours Work difficulties Work-related problems Accommodations Negative work events	**T:** PwMS with low conscientiousness. **I:** Psychotherapy with use of humanistic therapy techniques. Baseline treatment session (60 min): information about conscientiousness, its components, its relationship with health outcomes, and how it can decline in some individuals with MS. Formulation of SMART goals. After this session, participants were provided with the conscientiousness-coach app for smartphone and instructed on its use. Booster sessions (45 min) at weeks 4 and 8 to discuss accomplishments and achievements related to its goals and tasks + weekly telephone contact (1–2 min). Post-treatment interview at week 12. **C:** waitlist control	**N** **=** 11 **Age M(SD):** 45.1(8.6) **Women:** 81.7%, **Men:** 18.3% **Years of education M(SD):** 15(2.3) **Ethnicity:** NR **MS type:** 100% RRMS in intervention group, 83.3% RRMS in control group **Disease duration M(SD):** 13.9(4.6) years **(Neuro)psychological problems:** NR **Employment situation:** 100% employed. Hours per week M(SD) I: 34.6(10.9), C: 29.3(11.0)	The treatment group reported significantly more positive work outcomes relative to controls at follow-up (β = NR, 95% CI: NR, *p* = 0.028). Improved work outcomes for the treatment participants included increased work accommodations, such as flexible work hours, and promotions to positions of increased salary/responsibility. No significant effect of group or time was observed for MSWDQ-23
van den Akker et al., 2017^41^, Netherlands	RCT 52 weeks	Impact on participation and autonomy (IPA): work and education subscale	**T:** Ambulatory PwMS and severe MS-related fatigue, without depression or severe comorbid disorders. **I:** 12 sessions of individual face-to-face cognitive behavioral therapy spread over a 4-month period (8 sessions in the first 2 months, 4 sessions in the last 2 months) **A:** protocolled treatment by an experienced MS nurse that included three consultations of 45 min over a 4-month period.	**N** **=** 91 **Age M(SD):** 48.5(13.2) **Women:** 76.9%, **Men:** 23.1% **Years/level of education:** NR. **Ethnicity:** NR **MS type:** 73.6% RRMS, 11.0% PPMS, 13.2% SPMS, 1.1% other, 1.1% unknown **Disease duration median (IQR) years:** 6.7(2.1–14.2) **(Neuro)psychological problems:** 100% fatigue, no clinical depression **Employment situation:** 46% employed, 43% unemployed, 3.3% studying, 4.4% retirement, 3.3% unknown. 19.8% disability benefit.	Significant between-group effect on the IPA work and education domain after 26 weeks (β = −0.34, 95% CI: −0.66 to −0.03). No significant differences in other work-related outcomes between I and A groups.
De gier et al., 2024^36^, Netherlands	RCT 1 year	Work and social adjustment Scale (WSAS)	**T:** Ambulatory PwMS and severe MS-related fatigue, without depression or severe comorbid disorders. **I:** A blended booster program called ‘MS Stay Fit’ consisting of online booster modules and two booster video consultations with the same therapist as in the preceding cognitive behavioral therapy (CBT) at 2 and 4 months, after finishing the 20-week CBT for MS-related fatigue. The booster program was aimed at preventing fatigue relapse, and eventually reaching treatment goals that were not fully met during the first 20 weeks of treatment. **C:** No booster program, treatment ended after the last treatment session at 20 weeks.	**N** **=** 126 **Age M(SD):** 44.9(-) **Women:** 77%, **Men:** 23% **Education level:** 7.1% low, 41.3% medium, 51.6% high **Ethnicity:** NR **MS type:** 79.4% RRMS, 0.5% SPMS, 9.5% PPMS **Disease duration median(min-max) years:** 7 (0–41) **(Neuro)psychological problems:** 100% fatigue, 0% anxiety or depression. **Employment situation:** NR	No significant differences in WSAS between I and C groups.
Blikman et al., 2017^44^, Netherlands*	RCT 1 year	Impact on participation and autonomy (IPA): work and education subscale	**T:** PwMS with primary MS-related fatigue. **I:** Individual energy conservation management aimed at promoting a positive attitude aimed at decision-making and the optimum use of the available energy to fit the unique needs of each individual. 12 individual face-to face sessions of 45 min with an occupational therapist over 4 months. **C:** Information-only control condition. Three individual face-to-face MS nurse consultations of 45 min each by over 4 months.	**N** **=** 86 **Age M(SD):** 47.2(11.3) **Women:** 74.4%, **Men:** 25.6% **Years/ level of education:** NR **Ethnicity:** NR **MS type:** 74.4% RRMS, 7.0% PPMS, 16.3% SPMS, 2.3% unknown **Disease duration median(Q1–Q3):** 7(3.4–15.7) years **(Neuro)psychological problems:** 100% fatigue, subjective cognitive problems MFIS (range 0–40) baseline M(SD) I: 19.9(7.6), C: 18.2(8.8) **Employment situation:** NR	No significant differences in work-related outcomes between I and C groups.
Diamond et al., 2025^37^, Australia+	RCT 12 months	Work and social adjustment scale (WSAS)	**T:** PwMS **I:** Combined cognitive behavioral therapy (CBT) with cognitive training. Weekly two-hour group sessions facilitated by clinical neuropsychologists. Additional training: participants were also encouraged to use the BrainHQ cognitive training program for at least 20 min, three times per week outside of session. C: Usual care group.	**N** **=** 105 **Age M(SD)**: 46.7(11.5) **Women:** 74.3%, **Men:** 25.7% **Years of education M(SD):** 14.9(2.63) **Ethnicity:** NR **MS type:** 74.3% RRMS, 5.7% PPMS,1.0% PRMS, 19.0% SPMS **Disease duration M(SD) years:** 15.2(12.8) **(Neuro)psychological problems:** 74.3% no depression **Employment situation:** NR	No significant differences in WSAS between I and C groups.

MS: multiple sclerosis; PwMS: people with MS; RCT: randomized controlled trial; RTW: return to work; RRMS: relapsing-remitting MS; PPMS: primary progressive MS; PRMS: progressive relapsing MS; SPMS: secondary progressive MS; OT: occupational therapist; IPA: impact on participation and autonomy; CBT: cognitive behavioral therapy; T: target group; I: intervention group; C: control group; A: alternative intervention group; M: mean; SD: standard deviation; NR: not reported; *: primarily aimed to improve work outcomes; +: found in updated search.

Moss-Morris et al.^38^ compared the effectiveness of a nurse-led cognitive behavioral therapy program to supportive listening for improving adjustment in the early stages of MS. Participants in the cognitive behavioral therapy program scored lower on the work and social adjustment scale (β = −0.22, 95% CI: −4.37 to 0.84), indicating that cognitive behavioral therapy may result in less impairment in functioning in terms of work, home management, social leisure, private leisure, and personal or family relationships. Lincoln et al.^40^ evaluated the effectiveness of a group cognitive rehabilitation program for improving attention and memory problems in people with MS, and found no significant differences in employment status between groups. Fuchs et al.^34^ studied the effectiveness of an application-facilitated behavioral intervention for improving adaptation to low conscientiousness and, in turn, improving employment. Participants who used the app reported significantly more positive work outcomes including increased work accommodations, such as flexible work hours, and promotions ((β = NR, 95% CI: NR, *p* = 0.028)) compared to the waitlist control group.

Van den Akker et al.^41^ evaluated the effectiveness of cognitive behavioral therapy to improve MS-related fatigue and participation. Participants who received 12 sessions of individualized cognitive behavioral therapy scored lower on the “work and education” domain of the impact on participation and autonomy questionnaire (β = −0.34, 95% CI: −0.66 to −0.03), indicating that they experienced less impairment in work and/or education than participants who received protocolled treatment consisting of three consultations of 45 min over a four-month period from an experienced MS nurse. De Gier et al.^36^ evaluated whether a blended booster program improves the outcome of cognitive behavioral therapy for MS-related fatigue on fatigue severity after one year. The intervention and control groups did not significantly differ on changes in the work and social adjustment scale. Notably, fatigue did significantly improve in both groups in this non-inferiority trial. Blikman et al.^44^ investigated the effectiveness of an individual energy conservation management intervention on fatigue and participation in people with primary MS-related fatigue. The intervention and control groups did not significantly differ on changes in the impact on participation and autonomy questionnaire “work and education” subscale. Diamond et al.^37^ evaluated the effects of a combined group-based cognitive behavioral therapy and cognitive training on cognitive and psychosocial outcomes. The intervention and control groups did not significantly differ on changes in the work and social adjustment scale.

One study^43^ evaluated the effectiveness of an exercise intervention on work outcomes (Table 3). Heine et al.^43^ evaluated the effectiveness of aerobic training on MS-related fatigue and societal participation in ambulant patients with severe MS-related fatigue. There were no group differences on changes in the impact on participation and autonomy questionnaire “work and education” subscale.

**Table 3.** Summary of exercise interventions.

First author, year, country	Study design Length of follow-up	Work-related outcome measure	Target group (T) Intervention (I) control (C)	Sample characteristics: demographic factors, MS features, neuropsychological/psychological problems, employment situation	Effectiveness of the intervention on employment outcome(s)
*Exercise interventions*					
Heine et al., 2017^43^, Netherlands	RCT 1 year	Impact on participation and autonomy (IPA): work and education subscale	**T:** PwMS who are severely fatigued. **I:** 30 min aerobic interval training on an electro-magnetic cycle ergometer, three times a week, for 16 weeks. **A:** Three 45-min consultations with an MS nurse over a 16-week period.	**N** **=** 89 **Age M(SD):** 45.8(9.7) **Women:** 73.3%, Men: 26.7% **Years/ level of education:** NR **Ethnicity:** NR **MS type:** 73.0% RRMS, 9.0% SPMS, 18.0% PPMS **Disease duration median (IQR):** 8.0(2.0–15.3) years **(Neuro)psychological problems:** 100% fatigue, subjective cognitive problems MFIS (range 0–40) M(SD): 17.4(7.7) **Employment situation:** NR.	No significant differences in work-related outcomes between I and A groups.

MS: multiple sclerosis; PwMS: people with MS; RCT: randomized controlled trial; IPA: impact on participation and autonomy; T: target group; I: intervention group; A: alternative intervention group; M: mean; SD: standard deviation; NR: not reported.

Four studies evaluated the effectiveness of combined interventions^32,33,39,45^ on work outcomes (Table 4). There was a primary work-related outcome for two studies.^32,39^

**Table 4.** Summary of combined interventions.

First author, year, country	Study design Length of follow-up	Work-related outcome measure	Target group (T) Intervention (I) control (C)/ Alternative intervention (A)	Sample characteristics: demographic factors, MS features, neuropsychological/psychological problems, employment situation	Effectiveness of the intervention on employment outcome(s)
*Vocational + psychological*					
Stimmel et al., 2023^32^, US*	Quasi RCT 1 year	Employment status Current employment concerns Negative work experiences	**T:** Employed women with MS at risk of job loss. **I:** An in-person appointment where they received feedback and recommendations as well as a copy of the report on their comprehensive neuropsychological assessment (MACFIMS). A care-coordinator nurse then contacted these participants at approximately 1- and 6-months following feedback to offer assistance in completing the given recommendations. **A:** Feedback from their comprehensive neuropsychological assessment and treatment recommendations via a phone call with the psychologist. A copy of the neuropsychological testing report was provided to their neurologist and mailed to participants. They were encouraged to call the psychologist or their neurologist if they had further questions.	**N** **=** 138 **Age M(SD):** 43.7(10.5) **Women:** 100%, **Men:** 0% **Years of education M(SD):** 15.2(1.9) **Ethnicity:** I: 72.7% white; 6.1% African American; 9.1% Hispanic; 12.2% other, A: 73.9% white; 4.3% African American; 13.0% Hispanic; 8.7% other. **MS type:** NR. **Disease duration:** NR. **(Neuro)psychological problems:** fatigue I: 93.9%, C: 87%. Cognitive difficulties I: 36.4%, C: 39.1% **Employment situation:** working full-time 85%, part-time 15%.	No significant differences in work-related outcomes between I and A groups.
*Vocational + exercise*					
Arntzen et al., 2024^39^, Norway*	Pilot RCT 11 weeks	Work difficulties	**T:** People with mild to moderate disability due to MS. **I:** CoreDISTparticipation intervention consisting of a digital meeting with the MS nurse (40 min) and a clinical assessment by the physiotherapist (60 min). First 2–5 weeks 60 min two times per week, home training for 30 min. Weeks 7–10 60 min two times per week 4 weeks. Home training was encouraged two times per week for 30 min. **C:** Usual care group	**N** **=** 29 **Age M(SD):** 49.1(8.4) **Women:** 72.4%, **Men:** 24.1% **Years/ level of education:** NR **Ethnicity:** NR **MS type:** 89.7% RRMS, 6.9%PPMS, 3.4% unknown **Disease duration M(SD) years:** 11.2(9.5) **(Neuro)psychological problems:** NR. **Employment situation:** 82.8% employed, 13.8% unemployed, 3.4% unknown	No significant differences in work difficulties between I and C groups.
*Psychological + exercise*					
Alketbi et al., 2021^33^, Egypt	Pilot RCT 3 months	Work and social adjustment scale (WSAS)	**T:** PwMS identified as a case level of fatigue. **I:** Eight 50-min sessions of weekly cognitive behavioral therapy in addition to a cognition-targeted exercise program. **A:** Eight 50-min sessions of weekly cognitive behavioral therapy in addition to a standardized physiotherapy program, consisting of symptom-targeted exercises.	**N** **=** 60 **Age M(SD):** 41.5(5) **Women:** 85.0%, **Men:** 15% **Education level:** 61.7% university or higher, 5.0% high school, 33.3% junior high or less MS type: 95% RRMS, 5% SPMS **Ethnicity:** NR **Disease duration M(SD):** 11.5(3.5) years **(Neuro)psychological problems:** 100% fatigue **Employment situation:** 91.7% employed, 8.3% retired.	WSAS improved significantly for the I group (β = −3.68, 95% CI: −5.82 to −1.537) and this improvement was maintained at 3 months follow-up (β = −5.162, 95% CI: −7.40 to −2.923)
Prosperini et al., 2025^45^, Italy	RCT 16 weeks	Work productivity and activity impairment due to MS (WPAI:MS)	**T:** PwMS and impaired processing speed (SDMT score ≤5th percentile of Italian normative values) **I: “**Exergames” intervention consisting of **p**laying video games selected from the “Wii Fit Plus” package (with the Ninento Wii balance board) in a prespecified order. In the first 4 weeks only games targeting trunk and static postural control (“Zazen,” “Table Tilt,” “Ski Slalom”); in week 4–8, additional games requiring dynamic balance control (“Penguin Slide,” “Tightrope Walk,” “Soccer Head,” “Bubble Head”) were added; 10 min were allocated for each game. **A:** Active comparator: the adaptive COGNI-TRAcK (self-administered, intensive and monitored cognitive training), based on adaptive working load algorithms and procedures for intensiveness regulation. Three different types of exercises, each around 10 min per session. **C:** Sham intervention: the non-adaptive COGNI-TRAcK consisting of an algorithm implementing two low difficulty levels cognitive training alternating every day, regardless of the user's performance, thus ensuring a “placebo-analogue” intervention.	**N** **=** 102 **Age M(SD):** 50.1(10.4) **Women:** 59.8%, **Men:** 40.2% **Years/ level of education:** 23.6% primary, 47% secondary (high school), 29.4% college or university **Ethnicity:** NR **MS type:** 65.7% RRMS, 9.8% PPMS, 24.5% SPMS **Disease duration M(SD) years:** 15.1(8.5) **(Neuro)psychological problems:** 100% impaired information processing speed (≤5th percentile of Italian normative values) **Employment situation:** NR	Among WPAI:MS items, absenteeism was reduced in exergames compared with sham intervention at week 8 (f 2 = 0.04, *p* = 0.044), with a mean-adjusted difference of −6.1 points (95% CI −12.0 to −0.2), but not at week 16.

Work and social adjustment scale: WSAS; MS: multiple sclerosis; PwMS: people with MS; RCT: randomized controlled trial; I: intervention group; C: control group; A: alternative intervention; M: mean; SD: standard deviation; NR: not reported; *: primarily aimed to improve work outcomes.

Stimmel et al.^32^ evaluated the effectiveness of a neuropsychologically based employment intervention for preventing job loss in women with MS. The intervention and control groups did not significantly differ on employment status, employment concerns, and negative work experiences. Arntzen et al.^39^ evaluated the efficacy of a new, multidisciplinary intervention addressing sensorimotor function, physical activity, and unemployment on work difficulties in people with MS. There were no significant differences between groups. Alketbi et al.^33^ evaluated the effectiveness of a cognition-targeted exercise versus symptom-targeted exercise for MS fatigue. Scores on the work and social adjustment scale were significantly lower in the cognition-targeted exercise group (β = −3.68, 95% CI: −5.82 to −1.537), and this maintained at three months follow-up (β = −5.162, 95% CI: −7.40 to −2.923), indicating that a program combining cognitive behavioral therapy and cognition-targeted exercise may result in less impairment in functioning in terms of work, home management, social leisure, private leisure, and personal or family relationships. Prosperini et al.^45^ evaluated the effect of exergames (video games delivered by off-the-shelf commercial devices that require whole-body efforts and gross motor coordination within a non-immersive virtual bi-dimensional environment) as a stand-alone approach for people with MS and impaired processing speed. The absenteeism subscale from the Work Productivity and Impairment due to MS questionnaire was reduced in exergames compared with sham intervention at week 8 (f^2^= 0.04, *p* = 0.044), with a mean adjusted difference of −6.1 points (95% CI −12.0 to −0.2), but not at week 16. This indicates that exergames may temporarily reduce absenteeism, although the effect disappears after the intervention period ends.

## Discussion

We identified 16 studies, including four vocational, seven psychological, and one exercise intervention(s), and four combining two intervention types (vocational and psychological, vocational and exercise, and two times psychological and exercise) that related to work. There was considerable heterogeneity among intervention types and work outcomes measured (including work status and work difficulties), with only eight studies primarily aimed to improve work outcomes. In addition, these interventions focused on different sub-groups of people with MS depending on the focus of the intervention (e.g. severely fatigued people with MS).

Two out of eight studies specifically aimed at improving work outcomes showed positive effects (one focused on improving work outcomes by addressing conscientiousness, and one on improving job seeking), as did two out of six studies not directly focused on improving work outcomes (both aimed to improve fatigue). All studies reporting significant work-related effects showed improvements in self-reported workability and other behavioral or functional outcomes, rather than in work status or job retention.

We found contradictory evidence on the effectiveness of online modules to support work outcomes in people with MS. One study focusing on the job-seeking process, job-interviewing skills, and career development was effective in improving people with MS’ self-assessed vocational situation and several MS work difficulties questionnaire subscales.^20^ Another study by the same research group investigated a similar intervention but found no significant difference in effects after four months.^42^ A possible explanation for this contradictory evidence may be the use of different outcome measures and differences in study design, target group, and sample size. The interventions of these studies differed in their approach, as the second one was peer-led and targeted mostly unemployed people with MS, while the first one focused on those actively seeking work, or already employed at baseline. Some outcome measures used in the latter study may be especially relevant to employed people (MS work difficulties questionnaire), and indeed most effects were found for this questionnaire. The studies also differed in follow-up duration and sample size. Although the follow-up duration in the second study was longer (four months vs four weeks), its sample size was relatively small (n = 29) compared to that of the first study (n = 95). Given the accessibility and potential of online resources, further research with larger samples and adequate follow-up is needed.

One out of two psychological interventions with a significant effect on work outcomes was a psychotherapy intervention focusing on improving conscientious behavior^34^ because low trait conscientiousness is a particularly strong predictor of employment loss in people with MS.^46,47^ However, this study had a small sample size (n = 11) and short follow-up period (12 weeks) due to circumstances relating to the COVID-19 pandemic. To determine whether psychotherapy focused on increasing conscientiousness behavior is truly effective in improving work outcomes, larger studies with a sufficient sample size are necessary.

Of the five interventions that improved work-related outcomes, two specifically targeted fatigue^33,41^ with cognitive behavioral therapy, suggesting that improving fatigue may be a key mechanism for improving work outcomes. This finding aligns with previous research identifying fatigue as a significant correlate of work outcomes.^48,49^ Furthermore, findings from a qualitative study highlighted fatigue as the most important reason to leave work or reduce employment.^50^ Fatigue was found to exert a cascading effect, contributing to other symptoms such as cognitive functioning and vision loss.^51,52^ The cumulative burden of these symptoms may impact work functioning as well.

One intervention^45^ indicated that playing exergames effects absenteeism in people with MS and impaired processing speed. However, the effect was small and did not remain after the intervention ended. Playing exergames can temporarily improve MS-related symptoms such as fatigue^53^ which in turn can influence work outcomes, however absenteeism is influenced by multiple external and workplace factors.^54^ This may limit the long-term impact of such symptom-focused interventions on absenteeism. In addition, short-term improvements may also reflect a novelty or change-in-focus effect,^55^ whereby engagement in a new and stimulating activity temporarily enhances motivation or attention but diminishes over time. It is also possible that the 8-week intervention period was too short to produce a more robust or lasting effect. Further research on exergames interventions is needed to clarify these mechanisms.

The lack of significant effects on work outcomes in other studies may be due to small sample sizes, or insensitive outcomes measured (e.g. changes in employment status can take years to measure), or that the interventions were simply ineffective for such outcomes. Additionally, some outcome measures may not be suitable to identify changes to work outcomes. Among these outcome measures were the work and social adjustment scale^56^ and the work and education sub-scale of the impact on participation and autonomy questionnaire.^57^ Although these questionnaires reflect work outcomes in some way, these measures are part of generic health or functioning measures, incorporating work aspects, but do not clearly reflect actual participation in paid employment. Other work participation outcomes, such as employment status, working hours, or the MS work difficulties questionnaire may be more suitable to reflect employment changes. Furthermore, given that “working” is a complex phenomenon comprising several potentially interconnected individual, work-related, sociopolitical factors, it is important that the chosen work outcome measure is compatible with the focus of the intervention to be able to accurately assess the effectiveness of the intervention on this domain.

Overall, the risk of bias of the studies included in this review was considered high or moderate. This is partly due to the inability to blind those delivering or receiving the intervention, and the way outcomes were collected or assessed. Rehabilitation interventions using a randomized controlled trial design tend to be characterized by high risk of bias because these methods are more suitable for pharmacological interventions.^58^ Different criteria specifically applicable to non-pharmacological interventions are needed to accurately determine risk of bias in rehabilitation interventions.

Despite the negative impact of unemployment or work problems for people with MS, little progress has been made in developing and evaluating effective interventions that support work outcomes. The last (systematic) review in 2009^17^ included two studies, also included in the current review.^31,35^ In our review, we broadened the scope by including other intervention types; however, only a limited number of controlled studies have been published since. The wide range of interventions that were included in this review showed that most interventions for people with MS are designed for a specific sub-population. This may be explained by the heterogeneous nature of the disease. However, this limits the clinical applicability of these findings because the generalizability of the results remain unclear. The heterogeneous nature of MS and the differences in work contexts imply that personalized interventions may be more effective than general vocational rehabilitation interventions in enhancing work outcomes in this population. For studies that included a non-pre-stratified MS population, the manner in which participants were deemed eligible for a trial was not always specified which is a limitation of the original trials and by extension, this review. In addition, recent literature^59^ suggests that early interventions (providing support soon after diagnosis or before a crisis arises) may be more effective in improving work outcomes in people with MS. However, no studies focusing on early or personalized interventions for work outcomes were identified in this review. The effectiveness of an early personalized intervention on work outcomes is currently being studied in the “Don’t be late!” study.^60^

Furthermore, the development and approval of pharmacological interventions (disease modifying drugs) have influenced occupational outcomes in MS. A systematic review with meta-analysis found that unemployment in people with MS decreased, and that this decrease was significantly associated with the use of disease modifying drugs.^61^ This may be due to the possibility to control the disease and slow down its progression influencing the working capacity of people with MS.^62^ Therefore, the effects from older studies should be treated with caution compared to those from more recent studies, taking the use of disease modifying drugs into account. Additionally, despite evidence suggesting that job characteristics can be an important barrier for people with MS to improve work outcomes, only a limited number of included studies assessed the added value of interventions targeting workplace factors on work outcomes. Most included studies focused on treating symptoms related to MS instead.^63^

The strengths of this review are that it provides an exhaustive and reliable state-of-the-art overview of non-pharmacological interventions and their effectiveness on work outcomes. Unlike a systematic review, the methods followed in the scoping review provided sufficient flexibility to include a wider range of study designs and interventions. This study is also characterized by extensive rigor during the data screening, extraction, and synthesis, considering that at least two independent authors were involved for all stages of the review, and disagreements discussed with a third independent author. Conversely, comparison of effect sizes was not possible because (1) these were not reported for all included studies, and (2) due to the significant heterogeneity in both intervention types and reporting of outcome measures.

In sum, this scoping review showed that few interventions have been developed and tested to-date to support work outcomes for people with MS. Results from the available studies indicate that cognitive-behavioral therapy interventions for severely fatigued people with MS show promise in improving work outcomes, although further research is needed to substantiate these findings. In addition, studies showed variation in the type of non-pharmacological intervention, in the sub-groups of people with MS targeted by these interventions, and in the type of work-related outcomes assessed. This suggests that both the heterogeneous nature of MS as well as the diversity of work contexts create additional challenges in improving work outcomes for people with MS. Therefore, personalized interventions may ultimately prove more effective than general vocational rehabilitation interventions in enhancing work outcomes in this population.

**Clinical messages**

- Despite the importance of vocational rehabilitation, evidence on the effectiveness of non-pharmacological interventions aimed at improving work outcomes remains scarce.
- Findings indicate that cognitive-behavioral therapy interventions for severely fatigued people with MS show promise in improving work outcomes, but further research is needed to substantiate these findings.
- The heterogeneous nature of MS and variability in work contexts provide additional challenges when it comes to improving work outcomes in people with MS, indicating that personalized interventions may be more effective than general rehabilitation approaches.

## Supplemental Material

sj-docx-1-cre-10.1177_02692155261454943 - Supplemental material for Improving work outcomes in people with multiple sclerosis: A scoping review of non-pharmacological interventions Supplemental material, sj-docx-1-cre-10.1177_02692155261454943 for Improving work outcomes in people with multiple sclerosis: A scoping review of non-pharmacological interventions by Shalina RD Saddal, Blanca De Dios Perez, Alejandra Machado, Emilie Friberg, Roshan das Nair, Kathryn Radford, Joost G Daams, Hanneke E Hulst, Vincent de Groot, Karin van der Hiele and Frederieke Schaafsma in Clinical Rehabilitation
