# Supplementary material for: Improving work outcomes in people with multiple sclerosis: A scoping review of non-pharmacological interventions
Source: Clin Rehabil. 2026 May 25;40(11):1456–80. doi: 10.1177/02692155261454943 (PMC13736301; doi:10.1177/02692155261454943)
Supplement: Supplementary file 1 [file sj-docx-1-cre-10.1177_02692155261454943.docx]

**Supplementary Material**

**Ovid MEDLINE(R) ALL <1946 to March 31, 2026>
Search date: 1 April 2026**

**Ovid APA PsycInfo <1806 to March 2026 Week 4>
Search date: 1 April 2026**

**Web of Science
Search date: 1 April 2026**
